# Prevalence of Diabetic Retinopathy Among Patients With Diabetes and Its Correlation With Inflammatory Marker Levels in Tear Fluid

**DOI:** 10.7759/cureus.96692

**Published:** 2025-11-12

**Authors:** Neha Shibu Elenjickal, Austin Joju Akkara, Pradeoth M Korambayil, Prashanth Varkey Ambookan

**Affiliations:** 1 General Internal Medicine, Altnagelvin Hospital, Londonderry, GBR; 2 Plastic Surgery, Jubilee Mission Medical College and Research Institute, Thrissur, IND; 3 Diagnostic and Therapeutics, Zum Heilen Diagnostics and Therapeutics, Thrissur, IND

**Keywords:** diabetes mellitus, diabetic retinopathy, il-6, inflammatory markers, nf-κb, non-mydriatic fundoscopy, tear fluid, tnf-α

## Abstract

Background

Diabetic retinopathy (DR) is a leading microvascular complication of diabetes mellitus (DM) and a primary cause of vision loss worldwide. Markers such as NF-κB, TNF-α, and IL-6 have been proposed as potential early indicators on the basis of Inflammation playing a critical role in DR pathogenesis. This study aimed to determine the prevalence of DR among asymptomatic diabetic patients and evaluate correlations between tear fluid inflammatory markers and DR severity.

Methods

A cross-sectional observational study was conducted over two months at a tertiary care hospital in Kerala, India. Although an initial target of 120 participants was set, logistical and technical challenges in tear sample isolation limited recruitment to 50 asymptomatic diabetic patients. Fundus images were captured using a non-mydriatic handheld fundoscopy device with artificial intelligence (AI)-assisted grading of DR. Tear fluid samples, collected using Schirmer paper, were analysed for nuclear factor kappa B (NF-κB), tumor necrosis factor alpha (NF-α), and interleukin-6 (IL-6) via reverse transcription polymerase chain reaction (RT-PCR). Patient demographics, diabetes duration, comorbidities, and treatment history were documented. Associations between the presence of DR, clinical variables, and tear marker levels were analysed.

Results

DR was detected in 38% of participants, predominantly among patients aged >60 years and with longer diabetes duration. Insulin use and disease duration were significantly associated with DR, while age, gender, and oral antidiabetic use were not. Tear fluid inflammatory markers did not show a significant correlation with DR status. NF-κB was undetectable, and TNF-α and IL-6 levels were inconsistently elevated in DR-positive patients. Methodological factors, including tear sample over-dilution in 2 mL universal transport medium (UTM) and ocular surface conditions, likely contributed to these findings.

Conclusion

DR prevalence among asymptomatic diabetic patients is high, underscoring the importance of routine retinal screening. Non-mydriatic AI-assisted fundus imaging is an effective and practical tool for early detection. Tear fluid inflammatory markers, as measured in this study, were not predictive of DR, likely due to sample collection and amplification limitations. Future studies optimizing tear collection and RNA stabilization may establish reliable non-invasive biomarker-based screening, enabling earlier intervention and reducing vision-related morbidity in high-risk populations.

## Introduction

Today, one in 11 adults worldwide has diabetes mellitus (DM), 90% of adults have type 2 diabetes (T2DM), and China and India are the top two epicentres of the rapidly spreading global T2DM epidemic [1]. Diabetic retinopathy (DR) often remains asymptomatic until advanced stages, emphasizing the need for effective early detection. The inflammatory response is one of the main underlying molecular mechanisms involved in the pathophysiology of insulin resistance, DM, and its related complications [2]. DR, the most prevalent complication of DM [3], is projected to increase in prevalence from 103 million individuals in 2020 to 130 million by 2030 [4]. In India, DR affects 12.2% (95% CI: 10.4-14.1%) of the diabetic population [5]. Despite this considerable burden, systematic evaluation for DR is uncommon, and patients are frequently assessed only after the onset of ophthalmic symptoms, even when other diabetes-related complications such as diabetic foot, ketoacidosis, or nephropathy are evident. Notably, DR may precede the clinical diagnosis of T2DM, being present in approximately 7% of newly diagnosed cases, with incidence rising in proportion to disease duration. DR remains the leading cause of blindness in the working-age population and carries substantial socioeconomic consequences.

Routine retinal screening from the time of diabetes diagnosis facilitates timely intervention, while longitudinal follow-up enables early detection and treatment of vision-threatening DR, potentially preventing up to 98% of diabetes-related visual impairment [6]. In patients with macular oedema and proliferative DR, who are often asymptomatic, laser photocoagulation has been shown to reduce the risk of vision loss by 50% [7]. In this context, strengthening public health strategies to incorporate systematic screening and structured follow-up, alongside individual behavioural modification, is essential to mitigate the overall prevalence of DM and its complications [8].

The role of inflammation in the pathogenesis of DR is increasingly recognised. Patients with type 1 diabetes (T1DM) exhibit heightened inflammatory activity, whereas those with T2DM demonstrate chronic low-grade systemic inflammation; both groups display elevated circulating inflammatory markers [9]. These inflammatory mechanisms are implicated in both the initiation and progression of DR, underscoring their relevance as potential biomarkers for early disease detection.

In the present study, DR manifestations were evaluated using fundus imaging acquired with a non-mydriatic handheld fundoscopy, which provided artificial intelligence (AI)-based assessments of DR status. In parallel, inflammatory mediators, including nuclear factor kappa B (NF-κB), tumor necrosis factor alpha (NF-α), and interleukin-6 (IL-6), were measured to investigate their utility as objective molecular screening tools. TNF-α activates the NF-κB signalling cascade via its receptors TNFR-I and TNFR-II. Elevated soluble TNF receptor levels in the vitreous have been associated with DR, while a 10 pg/mL rise in serum TNF-α has been linked to a twofold increase in risk of proliferative DR. Similarly, IL-6 concentrations in the vitreous are elevated in both early and advanced stages of DR and serve as predictors of proliferative disease [10]. NF-κB, a central regulator of IL-6 gene activation, thus represents a critical marker of DR progression.

In India, Kerala is amongst the states with the highest prevalence of diabetes, with T2DM affecting approximately 10% of the adult population [11]. While screening is widely available in the private healthcare sector, it can be predominantly accessed only by affluent groups. Conversely, economically and socially disadvantaged populations rely primarily on the public health system, where access remains limited. This disparity highlights the urgent need for affordable, scalable, and accessible screening modalities to enable earlier diagnosis and intervention.

## Materials and methods

Study design and setting

This was an observational, cross-sectional study conducted over a period of two months at a tertiary care hospital in Kerala, India.

Sample size calculation

The required sample size was calculated using the formula: 4𝑝𝑞/ 𝑙^2^

where:

p = prevalence = 12.5 [12]

q = 100 - p = 87.5

l = precision = 6

Thus,

Sample size = 4 x 12.5 x 87.5 / 6^2^ = 120 

Accordingly, 120 patients were included in the study.

Participant selection

Inclusion criteria: Patients diagnosed with DM

Exclusion criteria: Patients presenting with overt ophthalmic symptoms suggestive of DR (e.g., floaters, blurred or fluctuating vision, scotomas, or vision loss) and patients with known ophthalmic conditions; patients with systemic diseases associated with tear film abnormalities and auto-immune (e.g., rheumatoid arthritis, systemic lupus erythematosus, Sjögren’s syndrome, thyroid disorders including Graves’ disease and Hashimoto’s thyroiditis, asthma, or allergies); patients who recently use (within the past three months) topical anti-inflammatory or antibacterial eye drops, or systemic immunomodulatory agents; and patients in whom handheld fundoscopy was not feasible due to discomfort or technical limitations.

Informed consent

All eligible participants were provided with an information sheet outlining study procedures and objectives. Queries were addressed, and written informed consent was obtained prior to enrolment.

Data collection

Demographic and clinical data, including comorbidities, diabetes-related complications, and treatment history, were collected using a structured proforma. Clinical examination followed.

Fundus examination was performed using a handheld, non-mydriatic, FDA-approved Remidio fundoscope. Images were processed using an AI-based platform to determine DR status. In cases of interocular discrepancy, the eye with the more advanced DR stage (as determined by the principal investigator) was selected for tear sampling.

Tear collection and molecular analysis

Initial protocol: Tears were collected using Schirmer Blu Touch strips (Madhu Instruments Pvt. Ltd., Delhi, India), positioned after eversion of the lower eyelid. Strips were removed once the 10 mm marking was wetted, sealed in labelled plastic covers (unique three-digit code, 000-120), and stored at 4 °C until analysis.

A total of 50 tear samples were collected using Schirmer strips. In the initial protocol, samples were processed directly without the use of universal transport medium (UTM); however, 26 samples failed reverse transcription polymerase chain reaction (RT-PCR) quality control, demonstrated by the absence of ACTIN amplification, indicating suboptimal RNA extraction from the strips.

To improve RNA recovery, the protocol was modified by transferring Schirmer strips to 3 mL of UTM and storing them at 4 °C until analysis. This approach, however, resulted in excessive dilution of tear RNA, compromising amplification efficiency. Subsequently, RNA extraction was performed from 200 µL aliquots of the UTM, which demonstrated marginally improved RNA yield and amplification consistency.

RNA was extracted using a magnetic bead-based method, and the concentration was measured spectrophotometrically. The extracted RNA was reverse-transcribed to cDNA, and multiplex quantitative reverse-transcription PCR (qRT-PCR) was performed using the PROGNOSEEZ PLUS Multiplex Real-Time PCR kit with gene-specific primers and probes. The targets included IL-6, TNF-α, NF-κB, and ACTIN (internal control), amplified using TaqMan chemistry according to the manufacturer’s protocol.

Statistical analysis 

All data were tabulated in Microsoft Excel (Microsoft® Corp., Redmond, WA) and analysed using Statistical Product and Service Solutions (SPSS, version 25; IBM SPSS Statistics for Windows, Armonk, NY). Associations between categorical variables were assessed using the chi-square test. A p-value <0.05 was considered statistically significant.

## Results

A total of 50 patients with DM were included in the study. The demographic characteristics, clinical variables, and inflammatory marker results are summarized below. Comparative analysis, by chi-square, was performed for key variables, including age, gender, co-morbidities, duration of DM, and antidiabetic therapy received between DR-positive and DR-negative groups.

Demographic profile

The demographic profile of the study population was evaluated to determine its relationship with the presence of DR.

Gender

**Figure 1:**
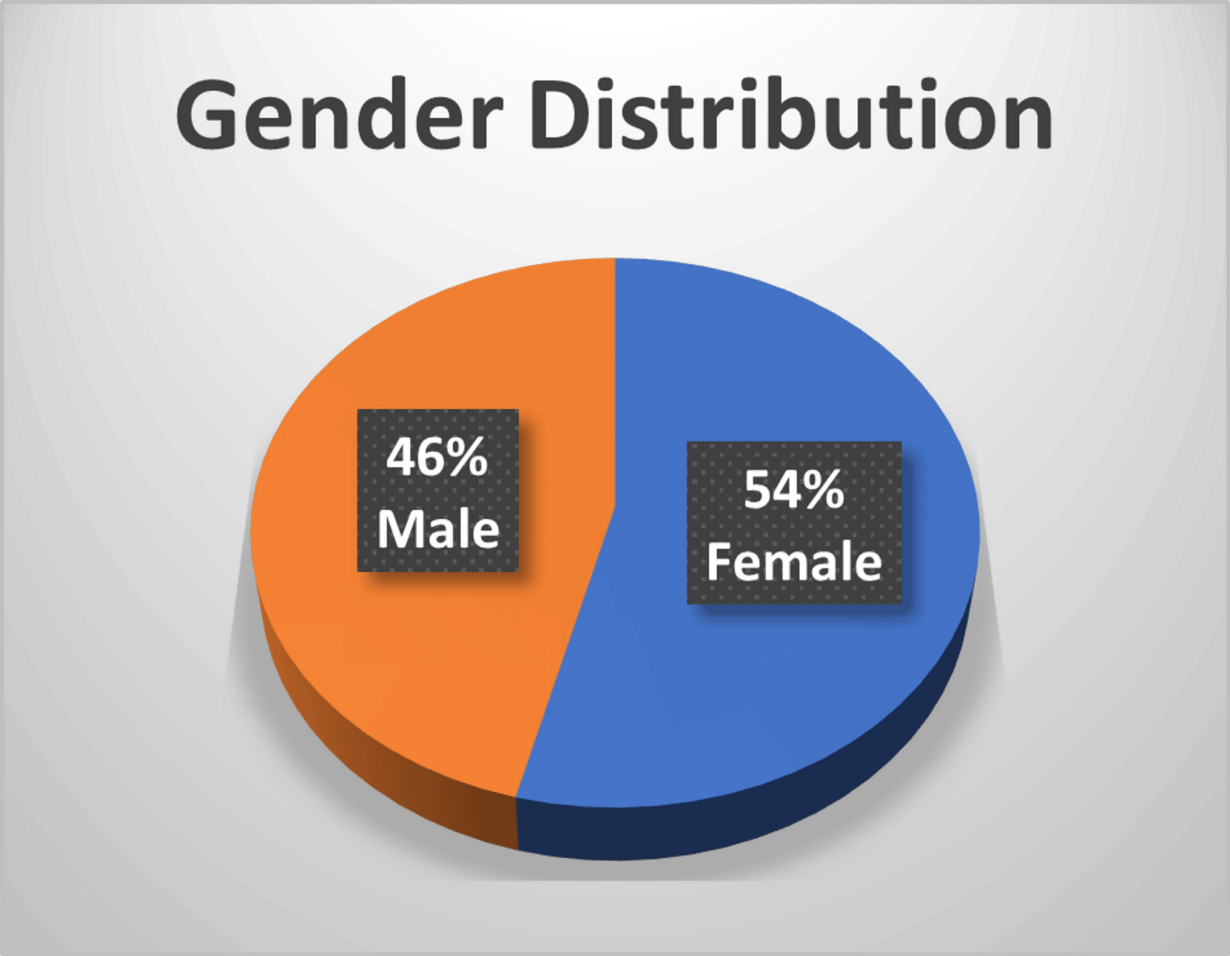
Gender distribution in the study

Data are presented as N (%). Among 50 patients with DM, 27 (54%) were female, and 23 (46%) were male. Among participants diagnosed with DR, eight (53%) were female, and seven (47%) were male (Figure 1).

**Figure 2:**
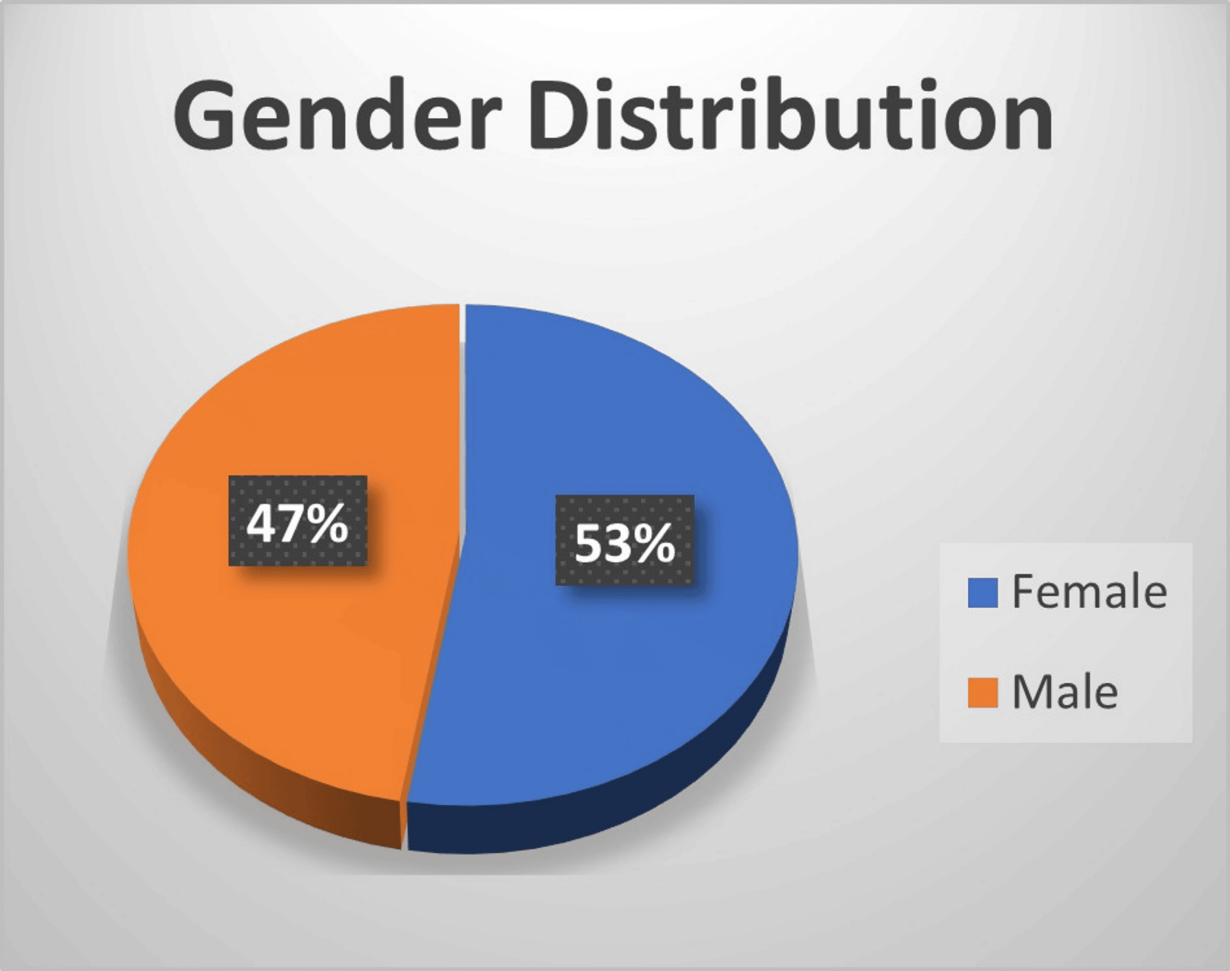
Gender distribution among oatients with diabetic retinopathy (DR)

Below data are presented as N (%). Among 19 patients with DR, 10 (53%) were female, and nine (47%) were male (Figure 2). 

Table 1 shows the number and percentage of male and female patients who were DR-positive and DR-negative. Among males (n = 23), 38.1% were DR-positive, and 60.9% were DR-negative. Among females (n = 27), 37.0% were DR-positive, and 63.0% were DR-negative.

**Table 1:** Gender-wise distribution of diabetic retinopathy among study participants (n = 50) No significant difference in DR prevalence was observed between males and females (p = 0.879). n: Number of patients, %: Percentage within the respective category, p-value: Calculated using the chi-square test to assess statistical significance, degrees of freedom: 1

Variables	Diabetic Retinopathy				Chi-square value (χ²)	P value
	DR-Positive		DR N-egative			
	n	%	n	%		
Gender						
Male	9	38.1	14	60.9	0.024	0.879
Female	10	37.0	17	63.0		

Age

The ages of the study participants ranged from 29 to 79 years, with a median age of 62 years (mean = 61.32, SD = 12.85) (Table 2). The most frequently observed age (mode) was 62 years. Statistical analysis using chi-square showed no significant difference in age distribution between groups (p = 0.32).

**Table 2:** Trend of age in patients

Particulars	Age
Mean	61.32
Median	62
Mode	62
Minimum	29
Maximum	79

**Figure 3:**
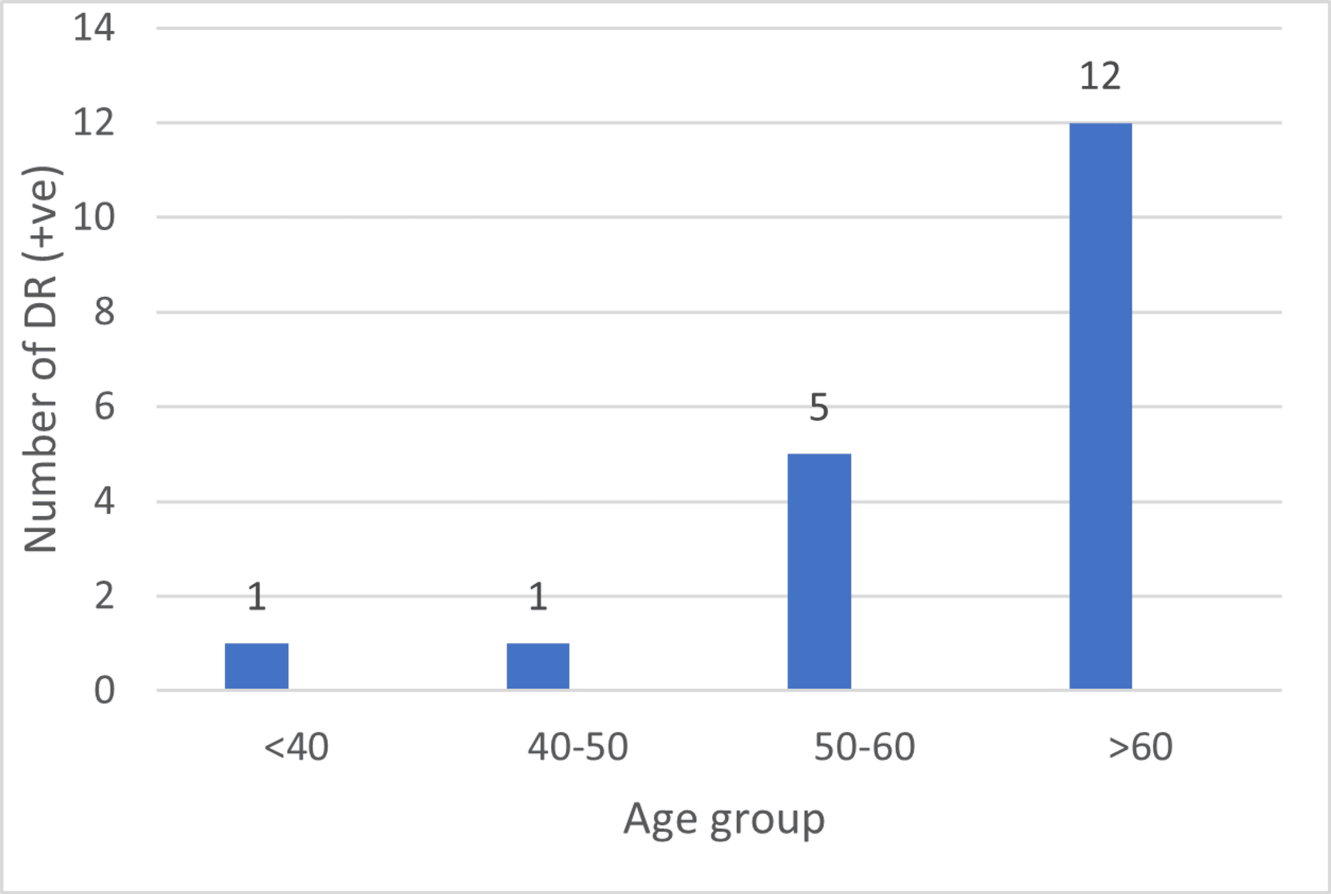
Distribution of diabetic retinopathy (DR +VE) patients by age (in years)

Out of the 19 patients who tested positive for DR, the majority (12 patients, 63.2%) were aged above 60 years. Five patients (26.3%) belonged to the 50-60 years age group, while only one patient each (5.3%) was observed in the <40 years and 40-50 years categories. The distribution indicates a higher prevalence of DR among older individuals (Figure 3). 

There was no statistically significant association between age and DR status (p = 0.32). Although a higher proportion of DR-positive patients was observed in the >60 years age group, this trend did not reach statistical significance (Table 3).

**Table 3:** Association between diabetic retinopathy and age groups (<60 years and ≥60 years) n: Number of patients, %: Percentage within the respective category, p-value: Calculated using the chi-square test to assess statistical significance, degrees of freedom: 1

Variables	Diabetic Retinopathy					p-value
	DR-Positive		DR-Negative		Chi-square value (χ²)	
	n	%	n	%		
Age						
<60 years	8	32	17	68	0.761	0.382
>60 years	11	19	14	56		

Comorbidities and diabetic complications

**Figure 4:**
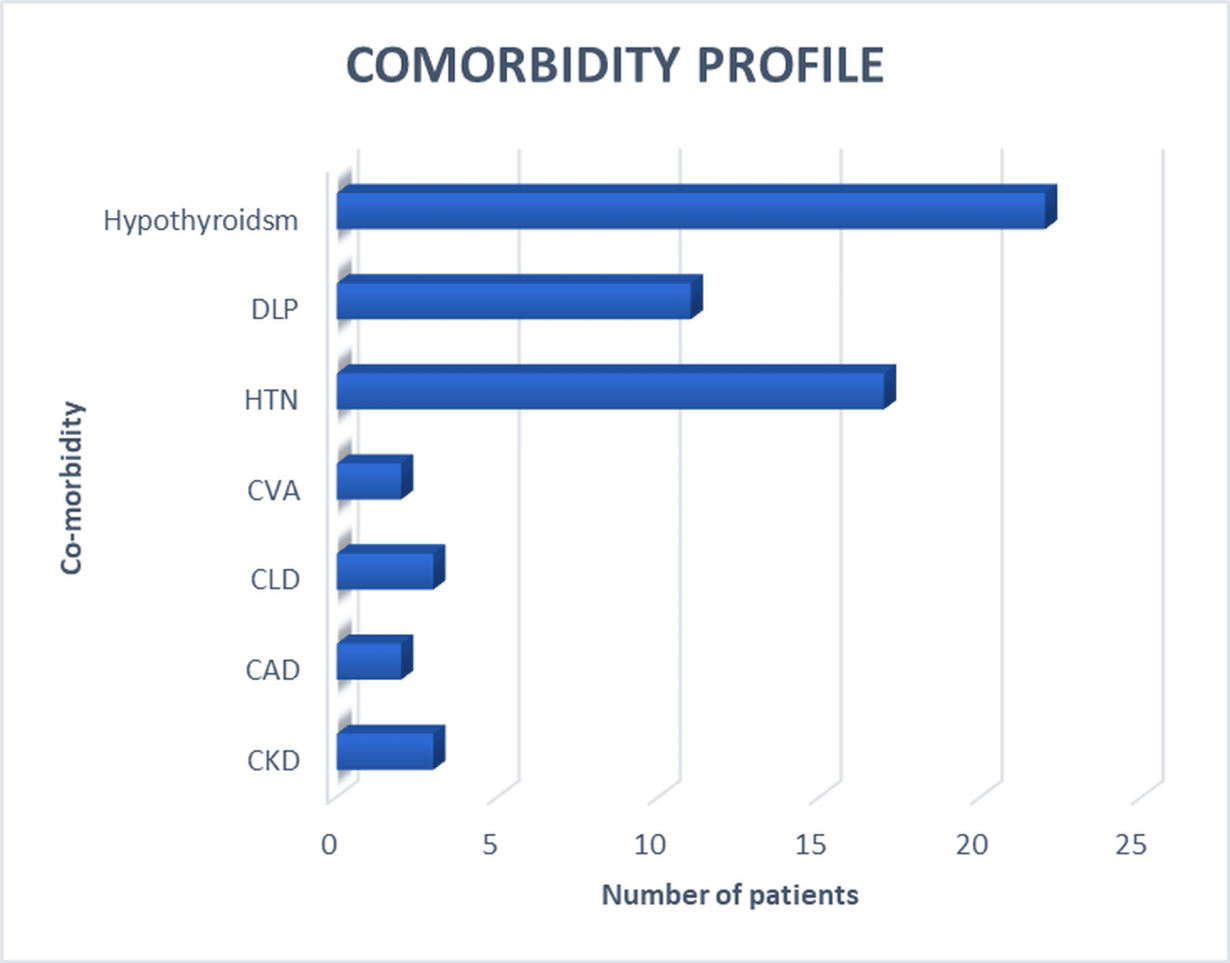
Comorbidities of the study participants HTN: hypertension; DLP: dyslipidemia; CAD: coronary artery disease; CVA: cerebrovascular accident; CKD: chronic kidney disease; CLD: chronic liver disease

The presence of systemic comorbidities, such as hypertension, dyslipidaemia, and cardiovascular disease, was documented among participants (Figure 4). Hypothyroidism was the most common comorbidity, observed in 22 patients (44%), followed by hypertension in 17 (34%) and dyslipidaemia in 11 (22%) patients. Chronic liver disease and chronic kidney disease were each present in three (6%) patients, while coronary artery disease and cerebrovascular accident were each reported in two (4%) patients.

Due to the limited sample size and availability of only grouped percentage data, regression analysis could not be performed. Instead, descriptive assessment was carried out to explore potential associations between comorbidities and DR. Although hypothyroidism and hypertension were the most frequent comorbidities, no distinct trend was observed between the presence of specific systemic conditions and DR grading.

**Figure 5:**
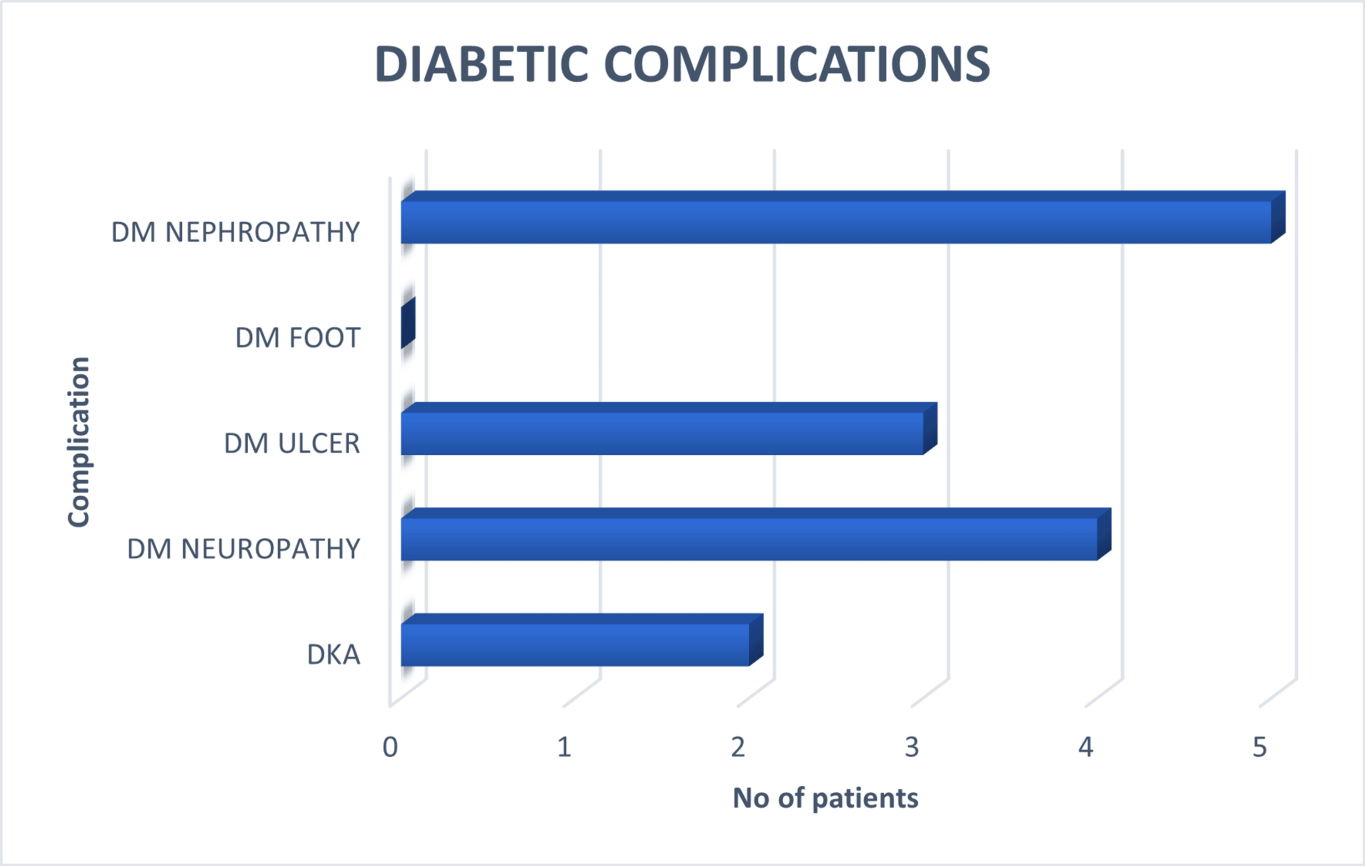
Known diabetic complications in study participants

Among the 50 patients included in the study, the prevalence of DR was 38%, representing the most frequent complication observed. Diabetic nephropathy was present in five patients (10%), diabetic neuropathy in four patients (8%), and diabetic foot ulcer in three patients (6%), while no cases of diabetic foot were recorded (Figure 5). Additionally, two patients (4%) had experienced diabetic ketoacidosis (DKA).

**Figure 6:**
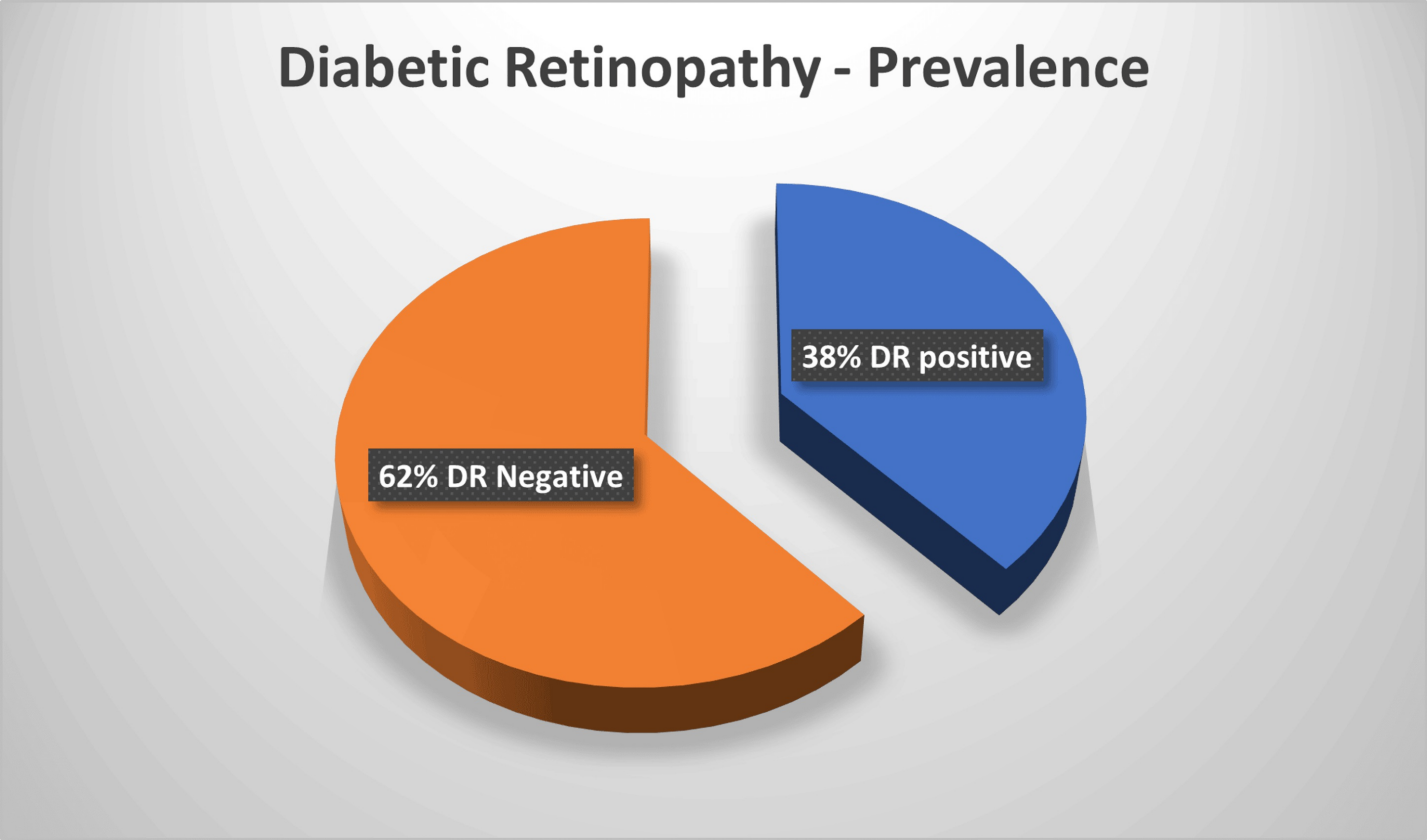
Distribution of diabetic retinopathy among study participants (n = 50)

Overall, DR constituted the predominant diabetic complication in this cohort. This pie chart (Figure 6) illustrates the prevalence of DR among the study cohort. Out of 50 patients evaluated, 38% (n = 19) were found to have positive findings for DR, while 62% (n = 31) showed no evidence of the condition. This distribution indicates that more than one-third of the studied diabetic population exhibited retinal changes suggestive of DR.

Duration of DM association with DR

The prevalence of DR increased with longer duration of diabetes (Table 4). Chi-square analysis demonstrated a significant association between disease duration and DR status (p = 0.001). Specifically, 80% of patients with ≥20 years of diabetes were DR positive, compared with 50% of those with 10-20 years and 17.9% of those with <10 years of disease, indicating a progressive rise in DR prevalence with increasing duration of diabetes.

**Table 4:** Association between the duration of diabetes and diabetic retinopathy (DR) The association between duration of diabetes and DR was evaluated using the chi-square test, revealing a statistically significant relationship (p = 0.001). n: Number of patients, %: Percentage within the respective category p-value: Calculated using the chi-square test to assess statistical significance, degrees of freedom: 1

Variables	Diabetic Retinopathy				Chi-square value (χ²)	p value
	DR-Positive		DR-Negative			
	n	%	n	%		
Duration of DM						
1-10 years	5	17.9	23	82.1	10.91	0.001
10-20 years	6	50	6	50		
≥ 20 years	8	80	2	20		

Anti-diabetic therapy associated with DR

To evaluate the influence of anti-diabetic therapy on the occurrence of DR, patients were stratified based on their use of oral anti-diabetic drugs (ADD) and insulin (Table 5). The prevalence of DR was compared between therapy groups to identify potential associations between treatment modality and DR risk.

**Table 5:** Association between anti-diabetic therapy (ADD) and insulin use) and diabetic retinopathy ADD: oral anti-diabetic drugs, Insulin Insulin therapy n: Number of patients, %: Percentage within the respective category p-value: Calculated using the chi-square test to assess statistical significance, degrees of freedom: 1

Variables	Diabetic Retinopathy				Chi-square value (χ²)	p value	
	DR-Positive		DR-Negative				
	n	%	n	%			
ADD use							
							Yes	17	37.8	28	62.2	0	1.00	
No	2	40	3	60									
Insulin use							
Yes	10	62.5	6	37.5	5.995	0.014	
No	9	26.5	25	73.5			

Inflammatory marker amplification

To standardize biomarker classification, Ct values were stratified into four categories: 12-20 = high positive, 21-28 = medium positive, 29-34 = low positive, and >34 = negative (Table 6). These thresholds were applied uniformly across all target genes (IL-6, TNF-α, NF-κB) to facilitate comparison of relative expression levels between samples.

**Table 6:** Classification of PCR Ct values for biomarker positivity PCR: polymerase chain reaction

Ct value	Interpretation
12-20	High positive
21-28	Medium positive
29-34	Low positive
34 above	Negative

Among samples that yielded successful amplification, IL-6 transcript was detected in 11 patients and TNF-α in 10 patients; NF-κB amplification was unsuccessful in all samples. For IL-6 (n = 11 amplified), low IL-6 positivity was observed in 10 patients, of whom 7/10 (58.3%) were DR-positive and 3/10 (41.7%) were DR-negative; medium IL-6 positivity was observed in six patients, of whom 5/6 (83.3%) were DR-positive and 1/6 (16.7%) were DR-negative. No high-positive or negative IL-6 categories were recorded among amplified samples. The overall chi-square test for IL-6 categories versus DR status yielded χ² = 0.36 (df = 1) and p = 0.55.

For TNF-α (n = 10 amplified), the distribution among amplified samples was as follows: negative TNF-α 4/4 (100.0%) DR-positive; low-positive 6/10 (60.0%) DR-positive and 4/10 (40.0%) DR-negative; medium-positive 1/1 (100.0%) DR-positive; and high-positive 1/1 (100.0%) DR-positive. The overall chi-square test for TNF-α categories versus DR status yielded χ² = 3.20 (df = 3) and p = 0.36.

Chi-square analyses were performed only on the subset of samples that produced reliable amplification for the respective gene; denominators for percentages therefore refer to the number of successfully amplified samples per biomarker rather than the full cohort of 50 participants. Table 7 presents the analysis of levels of inflammatory markers.

**Table 7:** Analysis of the levels of inflammatory markers NF-κB: nuclear factor kappa-light-chain-enhancer of activated B cells, TNF-α: tumor necrosis factor alpha, IL-6: interleukin-6

Variables	Diabetic Retinopathy				χ²	p value
	DR-Positive		DR-Negative			
	n	%	n	%		
Interleukin 6 (IL-6)						
Negative	0	0	0	0	0.36	0.55
Low positive	7	58.3	3	41.7		
Medium positive	5	83.3	1	16.7		
High positive	0	0	0	0		
Variables	Diabetic Retinopathy				χ²	p value
	DR-Positive		DR-Negative			
	n	%	n	%		
Tumor necrosis factor (TNF-α)						
Negative	4	100	0	0	3.2	0.36
Low positive	6	60	4	40		
Medium positive	1	100	0	0		
High positive	1	100	0	0		

Although inflammatory markers such as TNF-α and IL-6 were amplified in some samples, their expression levels were comparable between DR-positive and DR-negative individuals. The limited amplification success, particularly for NF-κB, may reflect low tear RNA yield or methodological constraints, warranting further optimization in future studies.

## Discussion

This study found that the duration of DM was the strongest factor associated with DR and that the prevalence of DR detected using AI-assisted non-mydriatic fundus imaging was 38%. This prevalence is notably higher than the 17.4% reported by Sivaprasad et al. [13]. Several factors may explain this disparity. A genuine increase in DR prevalence is possible, reflecting inadequate glycaemic control and inconsistent follow-up among patients with diabetes. Methodological differences may also contribute, as AI-based screening tools have an estimated false-positive rate of around 7% [14], particularly when image quality is suboptimal. Variations in sampling, population demographics, and the operational learning curve of fundus photography may have further influenced the results.

The present study also explored the potential role of inflammatory cytokines in tears as a non-invasive biomarker for DR. Chronic low-grade inflammation is now recognised as a central mechanism underlying insulin resistance, DM, and their microvascular complications. Cytokines such as TNF-α and IL-6 activate the JNK pathway, leading to serine phosphorylation of IRS-1 and disruption of insulin signalling, while NF-κB and other mediators exert similar inhibitory effects. Diabetic patients exhibit increased monocyte and macrophage activity, along with elevated circulating levels of TNF-α, IL-6, IL-1β, IL-18, MCP-1, resistin, PAI-1, E-selectin, and IFN-γ [2]. Building on this established pathophysiological link, the study evaluated tear-based inflammatory markers as a potential surrogate for retinal inflammation.

Prior research has shown that the tear proteome reflects both systemic and ocular pathology. Changes in tear composition have been documented following ocular insults such as cataract surgery, and proteomic analyses have revealed that tears can mirror pathological processes in retinal disease. Since the pioneering work of Herber et al. in 2000 [15], studies have consistently demonstrated that DR alters the tear proteome. Csősz et al. [16]. developed a mass-spectrometry-based approach revealing a general reduction in total tear protein content with DR onset, potentially due to impaired tear production or dilution, and identified several differentially expressed proteins, notably the inflammatory marker lactotransferrin. Along with lipocalin-1, lacritin, lysozyme-C, lipophilin-A, and immunoglobulin-λ chain, lactotransferrin was incorporated into a machine-learning model that successfully combined proteomic and imaging data for DR diagnosis. Other studies have identified reduced β-2-microglobulin levels and distinct glycomic profiles in the tears of DR patients compared with healthy controls. Together, these findings suggest that tear analysis offers a promising avenue for biomarker discovery in DR [17].

However, the current study did not demonstrate significant differences in IL-6 or TNF-α expression between DR and non-DR patients. This divergence from earlier reports may be explained by several methodological and biological factors. Technically, 26 of the 50 tear samples failed ACTIN amplification, reflecting challenges in RNA extraction from Schirmer strips in the absence of a UTM. Although later samples collected in UTM yielded better results, amplification of IL-6 and TNF-α was achieved in only 12, with high positivity in merely one DR-positive case. NF-κB was not amplified in any sample, contrasting with experimental work by Liang et al., which demonstrated NF-κB upregulation in diabetic rat retinas [18]. The use of 3 mL of UTM likely produced substantial dilution, potentially reducing biomarker concentrations by nearly 300-fold and hindering amplification. Moreover, most participants had mild or early DR, in which inflammatory activity may be minimal. Small tear volume, environmental contamination, and rapid turnover of tear fluid may have further reduced detectable cytokine levels.

These technical constraints limited statistical analysis, explaining why inferential testing, such as the chi-square test, could not be performed for TNF-α. Although descriptive trends were observed, the sample size was insufficient to draw robust conclusions. Future studies should therefore employ larger cohorts, improved extraction protocols, and more sensitive multiplex assays. Correlating tear cytokines with systemic parameters such as HbA1c, lipid profile, renal function, and serum cytokine levels may also help distinguish local from systemic inflammation. A longitudinal design could further clarify whether changes in tear biomarkers correspond to DR progression.

In the present study, 50 patients with DM were evaluated, comprising 27 females and 23 males, with a mean age of 63.5 years. A substantial proportion of the cohort had co-morbidities other than DM, including hypothyroidism (12%), chronic kidney disease (10%), hypertension (34%), cerebrovascular accident (4%), and dyslipidaemia (22%). Diabetic complications were also represented, with diabetic neuropathy (8%), diabetic ulcer (6%), and diabetic nephropathy (10%) being reported. Notably, none of the patients presented with diabetic foot disease or amputation, consistent with the premise that DR often precedes diabetic foot complications [19].

From a clinical and public-health perspective, DR remains a major cause of preventable blindness. Despite effective therapeutic options, delayed diagnosis continues to impede outcomes. Routine screening is often constrained by workforce limitations and the need for expert interpretation. The findings of this study reaffirm the utility of AI-assisted non-mydriatic fundus imaging as a practical screening strategy that reduces reliance on specialist evaluation. Although the hypothesis that tear-based cytokines could serve as a low-cost, home-based pre-screening tool was not supported, this approach remains conceptually attractive. In practice, stringent collection and transport conditions, along with the high cost of cytokine assays, currently limit the feasibility of mass screening. No significant correlation was identified between AI-detected DR and tear cytokine levels, suggesting that, with present technology, tear-based inflammatory analysis lacks diagnostic reliability.

While the concept of tear-based detection of IL-6 and TNF-α in DR is scientifically compelling, current methodologies appear inadequate for reliable clinical application. Nevertheless, AI-assisted fundus imaging demonstrated considerable promise in broadening DR screening capacity within primary-care settings. With methodological refinement and integration of systemic and ocular biomarkers, future research may yet establish tear analysis as a feasible adjunct in the early detection and monitoring of DR.

Limitations

This study was limited by a small sample size and technical challenges in tear sample processing. The use of 3 mL of UTM likely caused excessive dilution of tear biomarkers, impeding amplification; future studies should optimize by down-titrating UTM volume to improve concentration and detection efficiency. Additionally, missing systemic parameters such as HbA1c and lipid profile restricted correlation with systemic inflammation.

## Conclusions

Although effective treatments for DR exist, the rising prevalence of the disease underscores the limitations of current screening strategies, particularly in resource-limited settings where physicians face overwhelming workloads and inadequate training in fundoscopy. Non-mydriatic fundoscopy, supported by AI, demonstrates promise in alleviating this burden, though its use has an initial learning curve.

Our study explored the potential of inflammatory markers in tears as a non-invasive, objective screening tool for DR. Contrary to the hypothesis generated from earlier studies on inflammatory markers in other body fluids, no significant elevation was observed in patients with DR compared with controls, nor was a correlation found between tear fluid levels and fundus imaging. While the concept of a home-based, tear-based pre-screening test is attractive, practical challenges, including stringent participant selection, sample handling, and transport, significantly limit its feasibility. Additionally, the high costs associated with inflammatory marker assays present a considerable barrier to their use in large-scale population screening. Taken together, these findings suggest that, at present, inflammatory markers in tears do not provide a viable alternative to established DR screening modalities.
